# Contraction of the Foot in the Horse

**Published:** 1883-10

**Authors:** Thomas Walley

**Affiliations:** Principal of Royal (Dick’s) Veterinary College


### Art. XXV.—CONTRACTION OF THE FOOT IN THE HORSE.

BY THOMAS WALLEY, M. R. C. V. S.,
*Principal of Royal (Dick's) Veterinary College.*

On perusing the article on "Lameness," by Mr. McLellan, in the July number of your Journal, my attention was particularly attracted to his remarks upon the subject of contraction of the hoof; and as my experience and observations have led me to form opinions on the subject similar to those held by the writer I may be permitted to add a few remarks to those already made by him.

In the first place I may observe that to my mind there can be no question as to the alternate contraction and expansion of the hoof during progression, or, at least, of its posterior portions; and I think the fact can be easily demonstrated. But I must premise that the amount of expansion, or perhaps I ought to say whether expansion shall or shall not take place, will depend mainly upon the method of application of the shoe, *i. e.*, if the bearing surface of the shoe at the heels is made to incline inwards (as is too often the case), or if the shoe is nailed too far back and the nails clenched too firmly—no expansion can take place; on the contrary, the heels are constantly forced towards each other every time the horse puts his foot to the ground, and the proof of this is to be found in the fact that under such circumstances the part of the shoe with which the hoof has been in contact will be found polished by the friction, and not only polished, but in some cases actually indented, and the indentation and polishing will be found gradually shading off toward the inner edge of the shoe, while towards the outer edge the line is abrupt and well marked, showing that all movement has been in an inward direction.

If such a method of shoing is persisted in, the hoof gradually contracts and the horse goes lame.

Again, the hoof almost invariably contracts at the heel, or curves inward when the lateral cartilage becomes ossified.

The mechanism of expansion is, I think, as follows: The incidence, if weight passes through the column of bones, is in a downward and backward direction as the foot is placed on the ground, and the fetlock yields (also backward and downward), consequently the navicular bone and the flexor pedis perforans are forced in a similar direction, the result being, owing to the attachment of one division of the stellar navicular ligament to the inner aspect of the lateral cartilage, that the upper part of the cartilage is drawn inward and rendered tense, while, owing to the downward pressure exerted upon the tendon and fatty frog, the base of the cartilage, to some extent, the bulb of the heel to a greater, is forced outward, thus causing expansion of the postero-lateral portions of the hoof.

The tense condition of the cartilage under the circumstances stated is rendered very manifest by picking up the opposite part and throwing all the weight of the body upon the one under observation. That contraction is sometimes *a cause* of disease and lameness I am quite convinced, and the two following cases strikingly prove the proposition.

Many years ago I was asked to examine the feet of a pony with the object of giving evidence in a court of law as to the effect of long continued abnormal heat upon the hoof. The feet of the pony in question were good and the animal was perfectly sound when bought, he was put into a stable in close proximity to the boiler of an engine; in process of time the position of the fire-place was altered, the result being that the stable floor became very dry and hot; gradually the pony was observed to go short when in action; and the smithy noticed at each time of shoeing that all the hoofs were contracting. Ultimately the pony became useless.

In the second case I had blistered the fore legs of two chestnut cobs, 14.2 hands high, used as ladies pads, and had thrown them off work for six weeks during the summer; they were placed upon spent tan, and as the summer was excessively hot the tan became very dry, the man in charge neglecting to keep it damp; as a result both horses when they were taken off the tan came out lame, and on the shoes being removed they were both found to be the subjects of bi-lateral corn with marked contraction of the hoofs.

